# In Silico Identification of Potential Inhibitors of the SARS-CoV-2 Nucleocapsid Through Molecular Docking-Based Drug Repurposing

**DOI:** 10.1007/s44229-022-00004-z

**Published:** 2022-05-31

**Authors:** Rukhsar Afreen, Saleem Iqbal, Ab Rauf Shah, Heena Afreen, Lata Vodwal, Mohd. Shkir

**Affiliations:** 1 Department of Zoology, Gargi College, University of Delhi, New Delhi, 110049 India; 2 Molecular Endocrinology and Nephrology, CHU Research Center and Laval University, Quebec City, QC G1V 4G2 Canada; 3 Department of Biochemistry, University of Nebraska-Lincoln, Lincoln, NE 68503 USA; 4 Department of Computer Science, Jamia Millia Islamia, New Delhi, 110025 India; 5 Department of Chemistry, Maitreyi College, Chanakyapuri, New Delhi, India; 6 AFMOL, Department of Physics, College of Science, King Khalid University, Post Box-9004, Abha, 61413 Kingdom of Saudi Arabia

**Keywords:** SARS-CoV-2, Nucleocapsid protein, N-terminal domain, C-terminal domain, Molecular docking, Drug–protein interaction

## Abstract

SARS-CoV-2 is the virus responsible for the COVID-19 pandemic, and its effects on people worldwide continue to grow. Protein-targeted therapeutics are currently unavailable for this virus. As with other coronaviruses, the nucleocapsid (N) protein is the most conserved RNA-binding structural protein of SARS-CoV-2. The N protein is an appealing target because of its functional role in viral transcription and replication. Therefore, molecular docking method for structure-based drug design was used to investigate the binding energy and binding modes of various anti-N inhibitors in depth. The inhibitors selected were originally developed to target stress granules and other molecules involved in RNA biology, and were either FDA-approved or in the process of clinical trials for COVID-19. We aimed at targeting the N-terminal RNA binding domain (NTD) for molecular docking-based screening, on the basis of the first resolved crystal structure of SARS-CoV-2 N protein (PDB ID: 6M3M) and C-terminal domain (CTD) dimerization of the nucleocapsid phosphoprotein of SARS-COV-2 (PDB ID: 6WJI). Silmitasertib, nintedanib, ternatin, luteolin, and fedratinib were found to interact with RNA binding sites and to form a predicted protein interface with high binding energy. Similarly, silmitasertib, sirolimus-rapamycin, dovitinib, nintedanib, and fedratinib were found to interact with the SARS-CoV-2 N protein at its CTD dimerization sites, according to previous studies. In addition, we investigated an information gap regarding the relationships among the energetic landscape and stability and drug binding of the SARS-CoV-2 N NTD and CTD. Our in silico results clearly indicated that several tested drugs as potent putative inhibitors for COVID-19 therapeutics, thus indicating that they should be further validated as treatments to slow the spread of SARS-CoV-2.

**Supplementary Information:**

The online version contains supplementary material available at 10.1007/s44229-022-00004-z.

## Introduction

Since the emergence of the Delta variant of SARS-CoV-2 in May 2021, more than 155 million medically confirmed cases and approximately 3.24 million deaths occurred in more than 180 countries as a result of the COVID-19 pandemic [1]. The SARS-CoV-2/2019-nCoV novel coronavirus strain causes this respiratory disease [2, 3]. Repurposing of drugs has been a promising strategy at the forefront of strategies to address the continually growing number of COVID-19 cases [4–8]. In the current pandemic situation, drug repurposing should be considered a new avenue for the treatment of COVID-19 [5]. The coronavirus virion consists of structural proteins, namely spike (S), envelope (E), membrane (M), nucleocapsid (N) and, for some beta coronaviruses, hemagglutinin esterase [9]. The genomic structure of SARS-CoV-2 contains short untranslated regions and N-3′, E, M, 5′-replicase (rep)-S, identical to the genome structures of other coronaviruses [10]. As in other coronaviruses, the N protein is a crucial structural component of SARS-CoV-2 [11]. SARS-CoV-2 N has 90% sequence identity to the Severe Acute Respiratory Syndrome coronavirus N protein [12].

The N protein, which is naturally found within the virus, is the most conserved structural protein. It is required for viral replication and transcription, as it binds the viral RNA genome [13]. The N protein structure comprises an N-terminal RNA-binding domain (NTD), a C-terminal domain (CTD), and a naturally disordered central Arg/Ser-rich linker. For the SARS-CoV-2 N protein, each NTD molecule adopts a right-handed fist shape. The core sub domain consists of a five-stranded U-shaped antiparallel β-sheet with β4–β2–β3–β1–β5 topology, sandwiched between two short α-helices (α1 before the β2 strand and α2 after the β5 strand), and a protruding β-hairpin (β2′–β3′) is composed of mostly basic amino acid residues. New COVID-19 drug targets have been identified in the SARS-CoV-2-human interactome recently published by Gordon et al. [14]. Virtual screening and molecular docking have led to the identification of various inhibitors of SARS-CoV-2 [15].

Among the 332 interactions previously identified between viral and host proteins, most involve the innate immune signaling pathway [13, 16]. With this knowledge, researchers have identified an anti-N drug chain that shows excellent promise as a treatment for COVID-19 [17]. Through pathway analysis, a series of anti-N drugs with high potential to combat COVID-19 have been identified. Interestingly, some of the drugs target the N protein, which has been suggested to be a viable target for antiviral drug development [18]. The viral N protein targets the mTOR translational repressors LARP1, protein kinase CK2, and stress granule protein G3BP1/2. Viral replication is inhibited by stress granules and the host translation machinery [19, 20], whereas viruses suppress stress granules and use the host’s translation machinery for viral replication. Because of their roles in RNA genomic packaging, viral transcription, and replication, several recent studies have shown that coronavirus N proteins can be valuable antiviral drug targets [21–25]. Recently, studies have shown that the N proteins of coronavirus are valuable antiviral drug targets, owing to their roles in viral transcription, RNA genomic packing, and replication [26]. As C-terminal domain of N protein (CTD) is  involved in the self-assembly of N protein into a filament that is packaged into new virions, so structure-based molecular docking experiments have been performed on the CTD for the identification of possible inhibitors of N protein [17].

The first available crystal structures of N protein NTD (PDB ID:6M3M), abbreviated as N-NTD in this manuscript, and CTD (PDB ID: 6WJI) were published in late April 2020 [27]. Herein, these X-ray crystal structures were retrieved from the Protein Data Bank to conduct molecular docking to test the drugs recently described by Gordon et al. We considered all six FDA-approved drugs on the list, five drugs in clinical trials, and other compounds from the IUPHAR/BPS Guide to Pharmacology (2020-3-12) in our studies. This work focused on the identification of high confidence candidate drugs, and their effective roles in therapeutic interventions against COVID-19.

## Methods

### Molecular Docking

From the Protein Data Bank of RCSB (https://www.rcsb.org), we retrieved three-dimensional crystal structures of the SARS-CoV-2 N-NTD (PDB ID: 6M3M) and C-CTD (PDB ID: 6WJI), which were used as models for molecular docking analysis. PyRx, AutoDock Vina [28], and AutoDock Tools [29] were used for molecular docking analysis. SARS-CoV-2 C-CTD and N-NTD pockets at the binding site were selected with AutoDock Tools to create the grid box boundaries for docking [29]. For ligand preparation, such as charges, root detection, aromaticity, and hydrogen, the protein structure was converted to.pdbqt format (an accepted format for AutoDock Vina). Protein preparation for N-NTD and C-CTD proteins was performed by first adding the missing hydrogen atoms, then removing water and any other metal ions. PubChem (https://pubchem.ncbi.nlm.nih.gov) was used as a source to retrieve the structures of drugs. In PyRx, we converted all drug molecules from PDB to PDBQTs [29]. We took advantage of using PyRX rather than Vina because it can dock many more compounds in one session. The structure of proteins was pre-processed in PyMOL [30] by deletion of the chains, unoccupied binding sites, and ligands/water molecules from the protein structure. For docking purposes, the protein preparation was performed by addition of hydrogens and charges with the AutoDock tool. AutoDock Vina was used for molecular docking. The docking conformations of the top pose were obtained, and energy minimization of post-docking was performed in Discovery Studio (DS 3.531). Then PyMOL [30], Ligplot+ [31], and Discovery studio visualizer (Dassault Systèms BIOVIA 2017) were used to visualize and study the resultant receptor-ligand docked complexes.

### Drug–Target Network Construction

We used the STITCH (Search Tool for Interactions of Chemicals; www.stitch.embl.de) web server [32] to rationally select possible drug targets for drug–target network construction. The targets were identified on the basis of a network interaction score above 0.9.

## Results

### Binding Site Exploration

The CASTp Server was used to predict the binding sites by searching for CTD ligand-independent binding sites to perform molecular docking [33]. The CASTp Server identified a putative binding pocket with a volume of 1166 Å^3^ and a surface area of 907 Å^2^ (solvent accessible). The CTD binding pocket consisted of a coil region (335–349) at the C-terminus, and β2 (329–334), 259–264 (3_10_ helix), H4 (310–311, 314–318), 270, 274 (helix H1), H3 (301, 304–306), H2 (291, 292, 295–296), and 281–287 (turn and coil region between H1 and H5) residues (Supplementary Fig. S1). To predict the binding sites, we used two additional web servers, PrankWeb [34] and COACH [35]. Similar binding pocket residues with minor differences were also predicted with these two servers. Interestingly, both Protein–Protein Interaction Site Predictor (cons-PPISP) server and InterProSurf analysis indicated that most of the binding pocket residues were predicted to be potential dimeric interfacial residues [36, 37]. The results from these two web-servers reflected that the CTD dimerization residues are located in the coil regions adjacent to β1 (Arg319–Val324), β2 (Gly335–Leu339), H4 (Ser310–Ser318), and β2 (Gly328–Thr334).

### Molecular Docking

PPIs of SARS-CoV-2-human with 332 high-confidence and 69 candidate drugs have been identified to potentially treat COVID-19 in recent research performed by the Quantitative Biosciences Institute of University of California San Francisco.

**Fig. 1:**
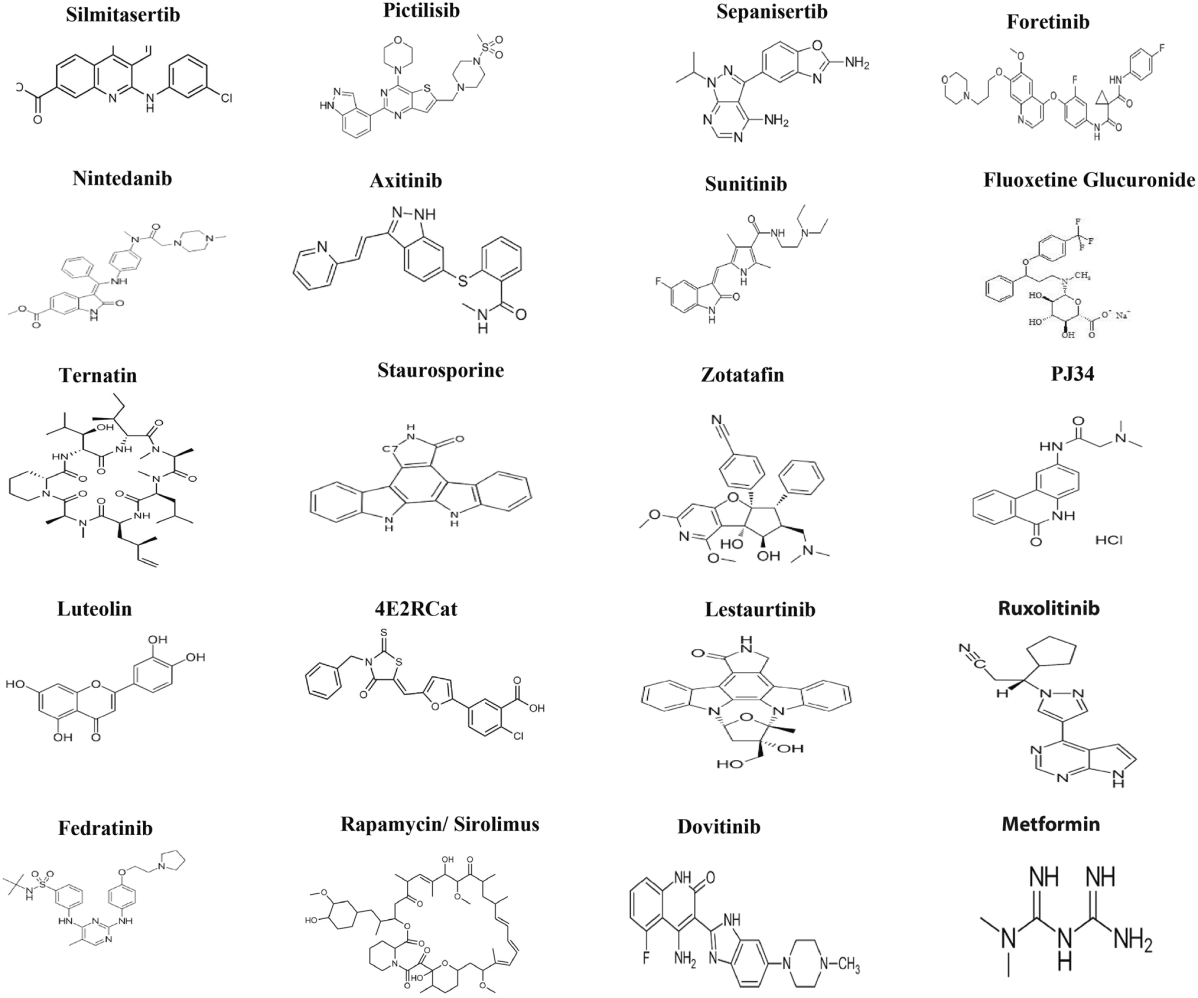
Structures of 20 miscellaneous drugs to combat COVID-19

Some of these drugs target the N protein and are already on the market, whereas others remain in phase 3 clinical trials. To verify the binding efficacy of compounds against the targets, we performed molecular docking analysis on the N-NTD (PDB ID: 6M3M) and C-CTD (PDB ID: 6WJI) proteins. On the basis of the binding affinity in kcal/mol, 20 potential drugs for COVID-19 were considered (Fig. 1). These drugs work in a variety of ways and include CK-2 inhibitors, mTOR inhibitors, translation inhibitors, SG inhibitors, and multi-targeted protein kinase inhibitors.

### Molecular Interactions Between the SARS-CoV-2 N-NTD and Drugs

**Fig. 2:**
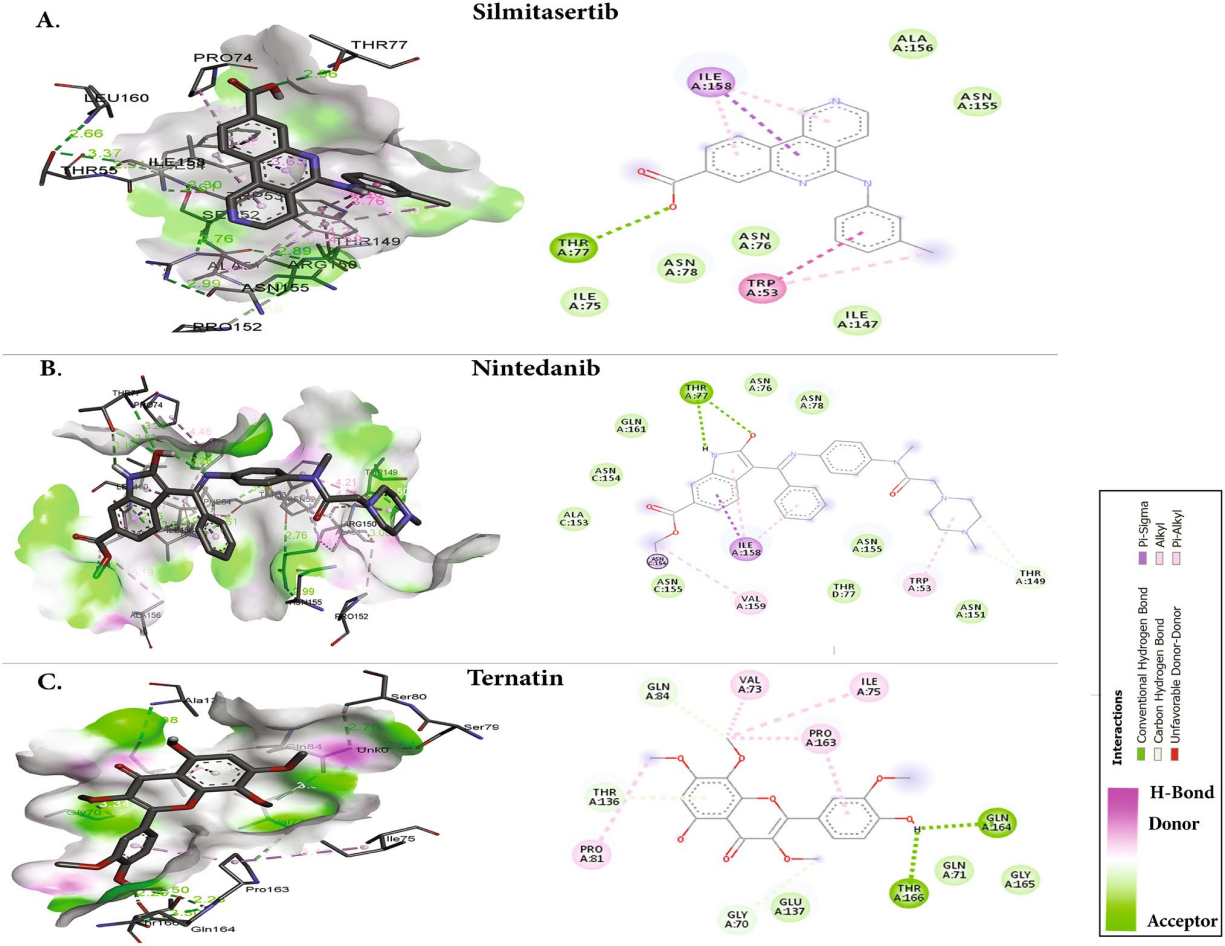
Molecular docking interactions and orientations of the top five anti-N drugs with the SARS-CoV-2 N-NTD. Docking interactions of **A** silmitasertib, **B** nintedanib, and **C** ternatin. The right panel shows a schematic representation of interactions of these drug molecules with the N-NTD. The protein residues and interactions are colored accordingly and provided in the figure

Virtual screening of compounds was followed by docking of potential high confidence drugs as well as candidate drugs, and the results were evaluated on the basis of binding affinity. Each of the 20 drugs were docked against the and evaluated according to their binding affinity against SARS-CoV-2 proteins (Table 1). Notably, the key residues involved in the RNA binding interactions, including S51, F53, R107, Y109, Y111, and R149 (in SARS-CoV-2 N-NTD numbering), are conserved, thus suggesting their potential for drug development. According to their binding affinities and the visual inspection of docked complexes for their ability to form hydrogen bonds and other interactions with the N-NTD, the best five molecules were chosen (Fig. 2). The compounds (and their corresponding binding affinities) were as follows: fedratinib (− 7.3 kcal/mol), luteolin (− 7.5 kcal/mol), nintedanib (− 8.2 kcal/mol), and ternatin (− 7.8 kcal/mol). Among all the compounds, silmitasertib showed the highest binding affinity (− 8.8 kcal/mol), exceeding that of the approved drug remdesivir (− 5.79 kcal/mol; [7]). Fedratinib bound with the least binding affinity (− 7.3 kcal/mol). Moreover, foretinib, dovitinib, axitinib, staurosporine, and sirolimus also exhibited strong binding, with binding energies of − 7.0 kcal/mol. As shown in Fig. 2A, silmitasertib, a casein kinase-2 inhibitor, showed the highest binding affinity with hydrogen-bonding residue Thr77, and hydrophobic interactions with Trp53, Ile75, Ile147, and Ile158, along with several van der Waals interactions with Asn76, Asn78, Asn155, and Ala156. Nintedanib, a tyrosine kinase inhibitor, formed two hydrogen bonds with Thr77, while the residues such as Trp53, Ala153, Ile158, and Val159 interacted hydrophobically, along with van der Waals interactions with three Asn residues (76, 78, and 155) (Fig. 2B**)**.

**Table 1:** Molecular docking results of 20 antiviral NTD of N protein inhibitors

Drugs	Binding affinity (kcal/mol)	Hydrogen bonds	Hydrophobic and van der Waals interactions
Silmitasertib	− 8.8	Thr77	Trp53, Asn(76,78,155), Ile(75,147), Ala156, Ile158
Nintedanib	− 8.2	Thr77, Thr149	Trp53, Asn(76,78,155), Ala153, Ile158, Val159, Gln161
Ternatin	− 7.8	Gly70, Gln84, Gln164, Thr136, Thr166	Val73, Ile75, Pro(81,163), Gln(71,165), Glu137
Luteolin	− 7.5	Gln59, Glu63, Asp64, Arg90, Arg93	Lys62, Leu65, Thr92, Arg94, Asp104, Leu105, Pro107, Trp109, Lys170,
Fedratinib	− 7.3	Thr58, Lys66	Ala(51,91,157), Ser52, Thr55, His60, Arg(93,108,150,174), Tyr110
Dovitinib	− 7.0	Thr77, Asn76, Ile147, Gln161	Trp53, Asn(78,155), His146, Ile158
Foretinib	− 7.0	Asn76, Ile147, Gln161	Trp53, Ile(75,147, 158), Thr(77,149), Asn78, Tyr113, His146, Gly148
Pictilisib	− 7.0	—	Trp53, Asn(76,78,154,155), Pro152, Ala(153,156), Ile(147,158), Val159, Gln161, Thr77
Axitinib	− 7.0	Asn154	Ile(75,147,158), Asn(76,78,155), Gln161, Val159, Thr77, Ser79, Trp53, Ala153
Staurosporine	− 6.9	Thr166, Leu162	Val159, Ala174, Leu(57,160,168), Pro163, Gly165, Gln164
4E2RCat	− 6.7	Gln161	Thr77, Ile(75,147,158), Trp53, Val159, Asn(76,78)
Rapamycin/sirolimus	− 6.7	Asp64, Thr167	Arg69, Pro(68,169), Trp133, Ile(131,132), Gly125, Asn127, Leu65, Glu63
Fluoxetine glucuronide	− 6.4	Ala156, Thr77, Ile75, Asn155	Asn(76,78,154), Ile(147,158), Trp53, Phe54, Gln161
PJ34	− 6.1	Tyr112	Ala(51,91,157), Tyr110, Arg(89,150), Thr55
Sapanisertib	− 6.1	Ile131, Lys128, Gly125	Lys66, Ile132, Trp133, Ala126, Asp(64,129), Leu65, Gly130, Asn127
Sunitinib	− 5.8	Pro81	Pro163, Thr(77,136,166), Glu137, Gly70, Gln(71,84), Ile75, Val73, Ser79, Asn76
Zotatifin	− 5.6	Thr166, Leu168	Leu(57,160,162), Tyr173, Ala174, Gln(161,164), Thr167
Lestaurtinib	− 5.6		Leu(57,160,162,168), Ala174, Gln(161,164), Thr166, Tyr173
Ruxolitinib	− 5.4	Asp64	Trp133, Gly125, Tyr124, Ile(131,132), Lys(66,128), Asp129
Metformin	− 5.3	Ile131, Asp129	Gly130, Arg90, Asp64, Trp109, Lys128, Ile132, Asn127, Ala126

**Fig. 3:**
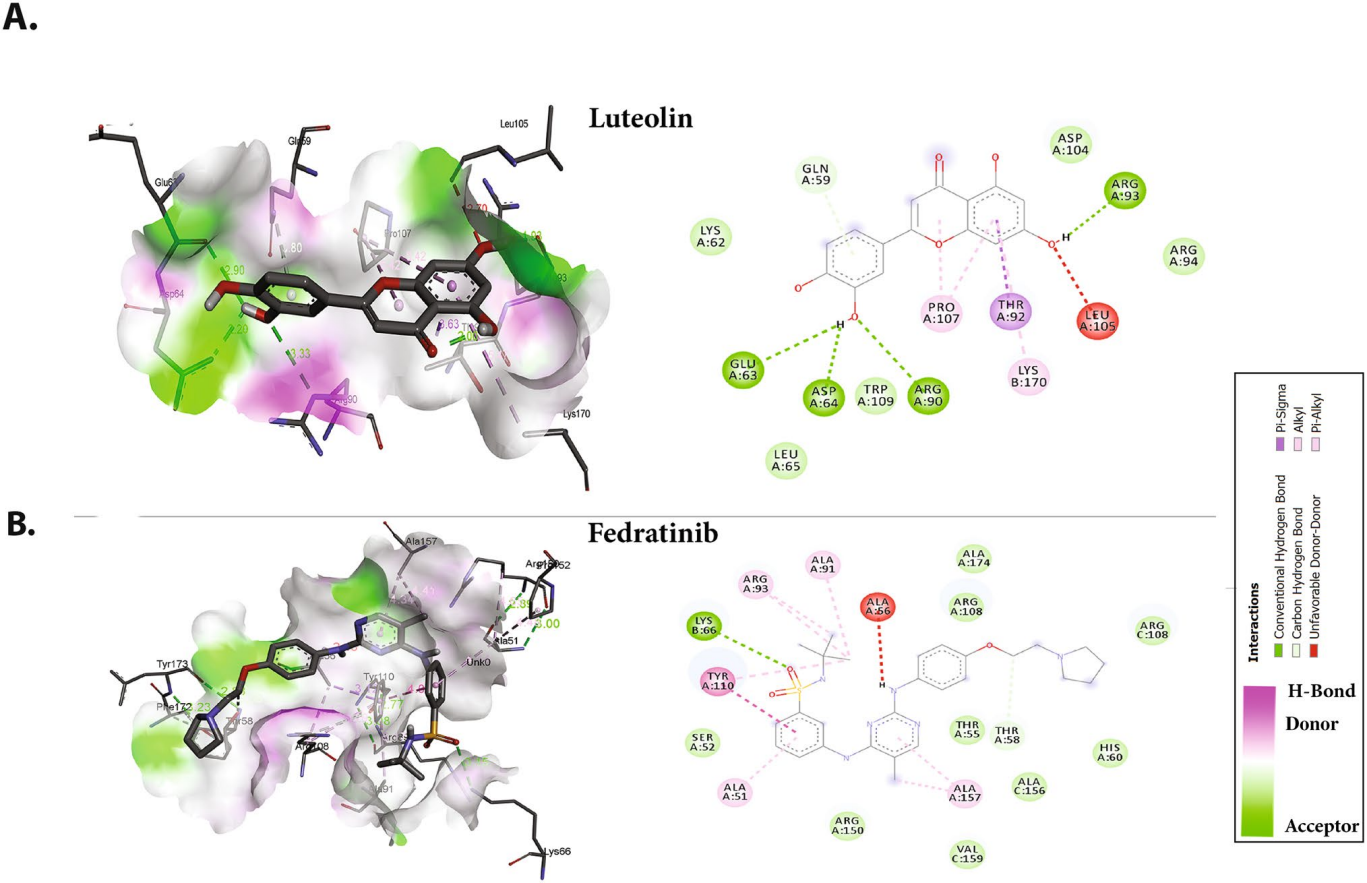
Molecular docking interactions and orientations of the top five anti-N drugs with the SARS-CoV-2 N-NTD. Docking interactions of **A** luteolin and **B** fedratinib. The right panel shows a schematic representation of interactions of these drug molecules with the N-NTD. The protein residues and interactions are colored accordingly and provided in the figure

Ternatin, a protein biogenesis inhibitor, formed five hydrogen bonds with Gly70, Gln84, Thr136, Gln164, and Thr166, along with hydrophobic interactions with residues including Val73, Ile75, Pro81, and Pro163 (Fig. 2C). Luteolin, an antiviral flavone, displayed hydrogen bonds involving Gln59, Glu63, Asp64, Arg90, and Arg93, along with hydrophobic and van der Waals interactions with Leu65, Thr92, Arg94, Leu105, Pro107, Trp109, and Lys170, displaying strong binding affinity (Fig. 3A).

The JAK2 inhibitor fedratinib interacts with the N-NTD through hydrogen bonds with Lys66 and Thr58, along the with hydrophobic interactions mediated by Ala51, Ser52, Thr55, His60, Ala91, Tyr110, Ala157, and Ala174. The binding is also characterized by van der Waals interactions with Thr55, Arg93, Arg108, and Arg150 (Fig. 3B). The fedratinib binding sites are similar to the RNA binding sites, thus suggesting that fedratinib binding may hinder the RNA binding of N protein.

Another anticancer drug, foretinib, interacted with the N-NTD via two hydrogen bonds involving Asn76, Ile147, and Gln161. Other key residues interacting with foretinib were Trp53, Ile75, Thr77, Asn78, Tyr113, His146, Gly148, and Ile158 (Supplementary Fig. S1**)**. The drugs dovitinib, staurosporine, and zotatifin also showed considerable binding affinity and interacted thorough hydrogen bonds, hydrophobic interactions, and van der Waals interactions (Supplementary Fig. S2).

Hence, the hydrogen bonding and hydrophobic interactions were the driving forces for binding, as indicated by the binding modes of top ranked drugs. The key residues of the N-NTD interacting with the drugs were Trp53, Ile75, Asn76, Thr77, Arg93, Arg94, Ala153, Ala156, Ile158, Val159, and Gln161, as shown by the results obtained from all drug docking calculations (Table 1). These residues are largely found in the turn region and are involved in ribonucleotide binding and protein–protein interfaces. Through the results of the cons-PPISP server, and InterProSurf analysis, residues in the β3 strand (Thr92–Arg96), the turn residues between β5 and β6 (Gly117, Pro118, Ala120, and Gly121), and the C-terminal tail (Asn151, Asn154, Ala157, Val159, and Gln161) were predicted to be potential PPI interfaces.

### Molecular Interactions Between the SARS-CoV-2 C-CTD and Drugs

**Fig. 4:**
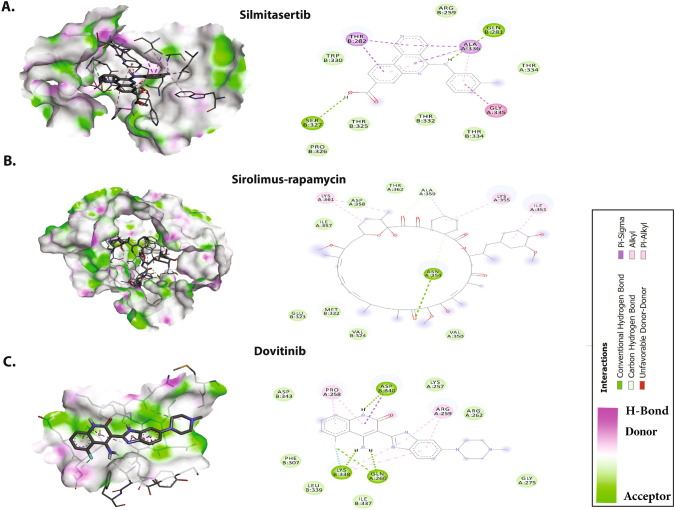
Molecular docking interactions and orientations of the top five anti-N drugs with the SARS-CoV-2 C-CTD. Docking interactions of **A** silmitasertib, **B** sirolimus-rapamycin, and **C** dovitinib. The right panel shows a schematic representation of interactions of these drug molecules with the C-CTD. The protein residues and interactions are colored accordingly and provided in the figure

All 20 drugs were docked against the C-CTD of N protein and rated according to their binding efficiency (Table 2). On the basis of their binding affinities and visual inspection of docked complexes for their ability to influence hydrogen bonds and other interactions with the C-CTD, the best five molecules were chosen (Fig. 4). The best five molecules (and their binding affinities) were fedratinib (− 8.2 kcal/mol), nintedanib (− 8.4 kcal/mol), sirolimus-rapamycin (− 9.3 kcal/mol), and dovitinib (− 8.6 kcal/mol); among these, silmitasertib showed the greatest binding affinity (− 9.3 kcal/mol).

**Table 2:** Molecular docking results of the top five antiviral CTD of N protein inhibitors

Drugs	Binding affinity (kcal/mol)	Hydrogen bonds	Hydrophobic and van der Waals interactions
Silmitasertib	− 9.7	GlaB:281, SerB:327	ThrB:282, AlaA:336, ArgB:259, AlaA:336, ThrA:334, GlyA:335, ThrB:334, ThrB:332, ThrB:325, ProB:326
Sirolimus-rapamycin	− 9.3	AsnA:354	IleA:357, LysA:361, AspA:362, AlaA:359, LysA:355, IleA:351, ValA:350, ValB:324, MetB:322, GluB:323
Dovitinib	− 8.6	AspB:840, LysB:338, GlnA:260	AspB:343, ProA:258, LysA:257, ArgA:259, ArgA:262, IleB:337, LeuB:339, PheB:307
Nintedanib	− 8.4	ArgB:262	GlyB:275, PheB:274, GlnB:283, ThrB:262, AspA:343, AspA:340, ProB:258, GlnA:349, PheA:307, IleA:337, GlnB:260, LeuA:339, ArgB:259, TrpB:330
Fedratinib	− 8.2	AspA:340, AspA:343	GlyB:275, ArgB:276, GlyB:284, GlyB:283, ThrB:282, PheB:274, LysA:338, TrpB:330, ArgB:259, GlnA:349, GlnA:306, GlnB:260, ProB:258, LeuA:339

As shown in Fig. 4A, silmitasertib, a casein kinase-2 inhibitor, showed the highest binding affinity and formation of hydrogen bonds with Glu281, and Ser327, and hydrophobic interactions with Thr282, Ala336, Arg259, Ala336, Thr334, Gly335, Thr334, Thr332, Thr325, and Pro326, along with several van der Waals interactions.

**Fig. 5:**
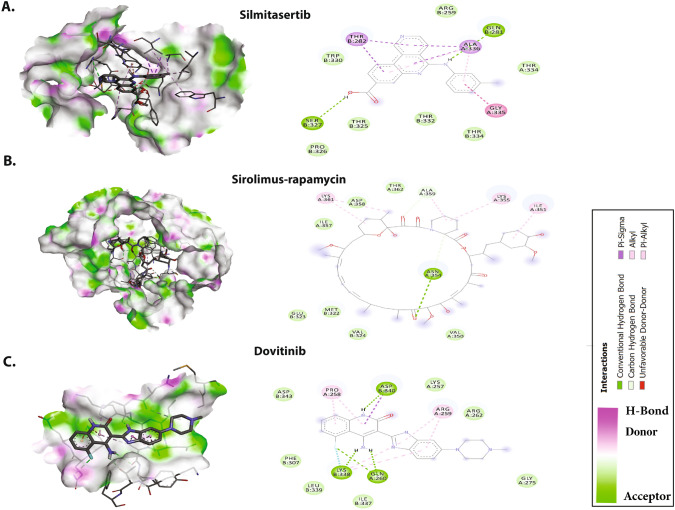
Molecular docking interactions and orientations of the top five anti-N drugs with the SARS-CoV-2 C-CTD. Docking interactions of **A** nintedanib and **B** fedratinib. The right panel shows a schematic representation of interactions of these drug molecules with the C-CTD. The protein residues and interactions are colored accordingly and provided in the figure

As shown in Fig. 4B, rapamycin (sirolimus), a potent immunosuppressive agent, blocks or inhibits cytokine-mediated signal transduction pathways during late T-cell cycle progression. This inhibition occurs through modulation of the function of a target protein. Rapamycin forms hydrogen bonds with Asn354 and hydrophobic interactions with Ile357, Lys361, Asp362, Ala359, Lys355, Ile351, Val350, Val324, Met322, and Glu323, along with several van der Waals interactions. Figure 4C shows that dovitinib is as anticancer drug that inhibits multiple kinases. It forms hydrogen bonds with the C-CTD through AspB:840, LysB:338, and GlnA:260. Dovitinib binds the C-CTD of N through hydrophobic and van der Waals interactions with Asp343, Pro258, Lys257, Arg259, Arg262, Ile337, Leu339, and Phe307. Nintedanib, a tyrosine kinase inhibitor, forms two hydrogen bonds with Arg262 and hydrophobic interactions with Gly275, Phe274, Gln283, Thr262, Asp343, Asp340, Pro258, Gln349, Phe307, Ile337, Gln260, Leu339, Arg259, and Trp330, along with van der Waals interactions with three Asn residues (76, 78, and 155) (Fig. 5A). The JAK2 inhibitor fedratinib interacts with residues such as Asp340 and Asp343 via hydrogen bonding, and forms hydrophobic interactions mediated by Gly275, Arg276, Gly284, Gly283, Thr282, Phe274, Lys338, Trp330, Arg259, Gln349, Gln306, Gln260, Pro258, and Leu339 (Fig. 5B).

### Interactions Between SARS-CoV-2 N and Human Proteins

**Fig. 6:**
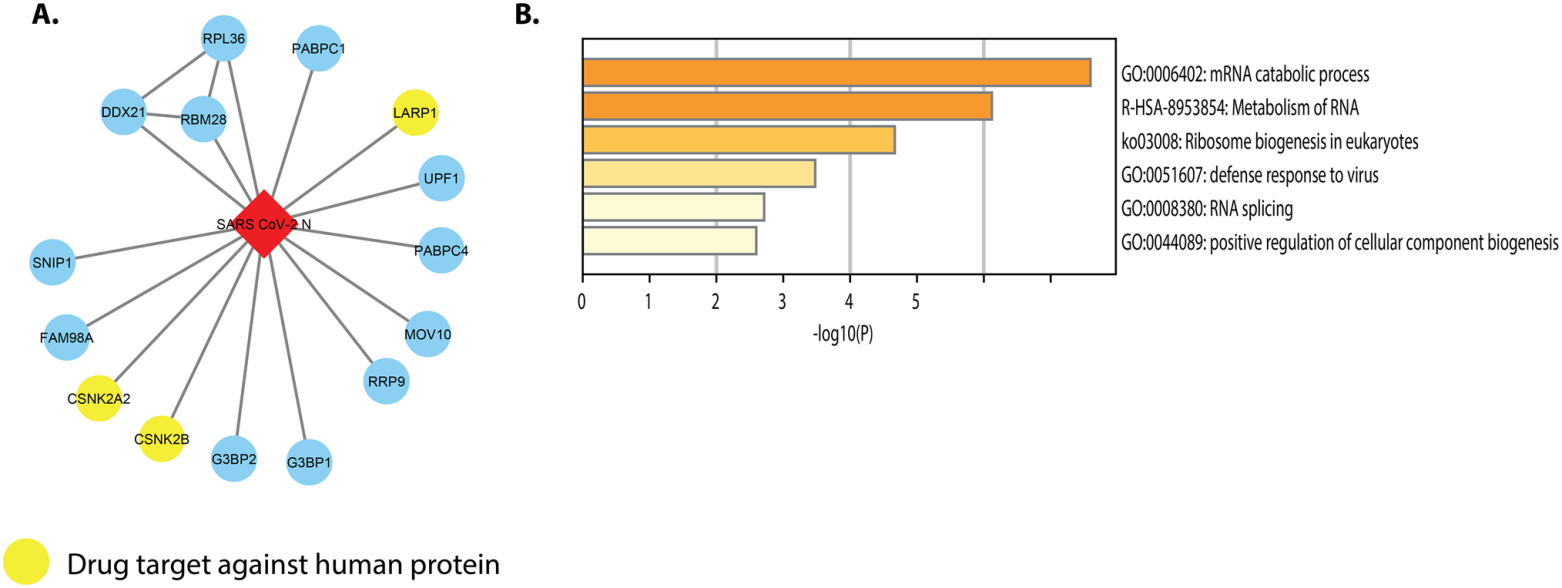
**A** Interactions between SARS-CoV-2 N and human proteins. **B** Bar graph of enriched terms across input gene lists, colored by *p*-values

Drug targets for drug repurposing were predicted by mapping the protein–protein interactions of SARS-CoV-2-human [14, 16]. Yellow color indicates human drug target interaction with SARS-CoV-2 (Fig. 6A). G3BP1, G3BP2, and LARP1 human proteins interact with N of SARS CoV-2 and are drug response proteins. Host proteins are involved in RNA splicing, viral defense, ribosome biogenesis in eukaryotes, metabolism of RNA, and mRNA catabolic processes (Fig. 6B).

### Identification of Potential Drug–Target Interactions

**Fig. 7:**
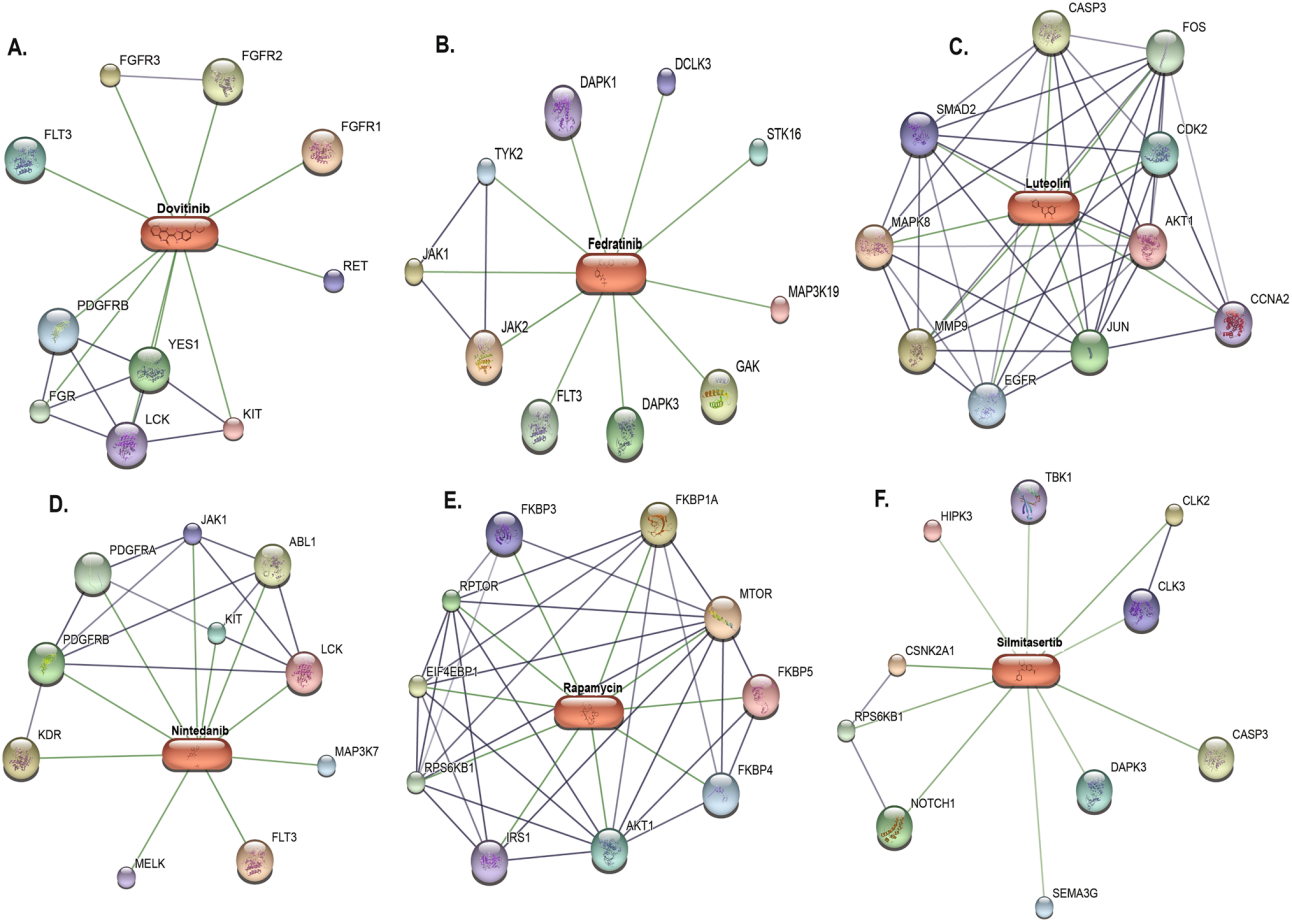
Drug–gene network constructed with STITCH. The edge width of protein–drug interactions is scaled according to the binding affinity between the drug and the protein. The drug–protein networks of **A** dovitinib, **B** fedratinib, **C** luteolin, **D** nintedanib, **E** rapamycin, and **F** silmitasertib

The STITCH database was used as a model for drug–protein interactions through a statistical approach. STITCH incorporates data from 2031 genomes on more than 5 million interactions between 430,000 chemicals and 9.6 million proteins. To predict protein–drug interactions, it primarily relies on keyword mining of the literature and experimental evidence. The likelihood that the expected relationship occurs is indicated by a confidence score (0–0.9). A confidence score of 0.9 or higher was used to discover the targets. To construct a network based on binding affinities (${K}_{i}$ of protein–drug interactions with thickness of edges between nodes, increasing as ${K}_{i}$ value increases), we used STITCH, which is based on STRING v10 [38]. Figure 7 shows the STITCH predictions for the drug–gene relationships among the top five strongly binding drugs. Some of these drugs had relatively fewer high affinity binding targets. Prominent proteins included YES1, RET, FGR, and FGFR1/2/3, which are involved in cancer and associated pathways as well as endocytosis, and dovitinib has been found to target some of these proteins (Fig. 7A). Fedratinib interacted with the JAK2/JAK1 proteins, which are part of the JAK-STAT signaling pathway (Fig. 7B).

Luteolin, a flavone, had a low affinity for the proteins CASP3, JUN, CDK2, FOS, and MAPK8, which are involved in the TNF signaling pathway and cancer (Fig. 7C). However, certain drugs had several predicted protein interactions and interacted via various pathways. For example, nintedanib targets the Ras signaling pathway, cancer, and cytokine–cytokine receptor interaction proteins MAP3K7, JAK1, PDGFRA/B, LCK, KIT, MELK, FLT3, and KDR (Fig. 7D). Similarly, sirolimus/rapamycin, a kinase inhibitor, interacts with many targets in the mTOR and AMPK signaling pathways, and with proteins involved in immunosuppression, including mTOR, FKBP1A, and FKBP5 (Fig. 7E). Silmitasertib interacts with CSNK2A1, a serine/threonine kinase protein involved in cell cycle progression, apoptosis, transcription, and viral infection (Fig. 7A). Another drug, foretinib, is involved in endocytosis and focal adhesions, and has similar targets to nintedanib and dovitinib.

## Discussion

As the RNA binding activity of N protein is essential for viral ribonucleoprotein formation and genome replication, blocking the RNA binding of the N-NTD has been demonstrated to be a potential treatment strategy. N protein is an essential RNA-binding protein with crucial roles in replication and transcription of viral RNA. An overall right-handed fold with a β-sheet core is found between loops, as revealed by recently solved crystal structures of the SARS-CoV-2 N-NTD (PDB ID: 6M3M) and C-CTD (PDB ID: 6WJI). The core region of the β-sheet consists of five antiparallel β-strands with a β6–β2–β5–β1–β7 topology flanked by a single short α-helix just before strand β2, and a protruding β-hairpin (β3 and β4) between strands β2 and β5. In addition, an NMR structure of the SARS-CoV-2 N-NTD in complex with RNA (PDB ID: 6YI3) suggested putative RNA binding sites of A51, T58, H60, R93, I95, L105, S106, R108, R150, and Y173. Moreover, the adenosine monophosphate (AMP) binding site has been structurally characterized in HCoV-OC43 N-NTD by Lin et al. [22]. N49, A51, S52, A56, R89, R108, Y110, Y112, and R150 compose the AMP binding site, as indicated by the structural superposition between the SARS-CoV-2 N-NTD and HCoV-OC43 N-NTD-AMP. Therefore, we extended our investigation by using structure-based molecular docking of N-NTD with different drugs to gain insights into the structural and molecular regions’ potential effectiveness in antiviral drug therapy. The investigated drugs included protein biogenesis inhibitors, anticancer compounds, antiinflammatory compounds, mTOR inhibitors, and stress granule modifiers. The docking results showed that 5 of the 20 drugs bound with strong binding affinity. Among N-NTD inhibitors, the key residues involved in binding were near the helix, i.e., Trp53, Ile75, Asn76, and Thr77; in the β3 strand, i.e., Arg93 and Arg94; and at the C-terminal interface, i.e., Ala153, Ala156, Ile158, Val159, and Gln161.

Silmitasertib is an antiviral drug that has been tested against the N protein of SARS-CoV-2, and found to block the CK2 and enhance SGs formation [39], thus inhibiting SARS-CoV-2 proliferation in vitro. Recently, Taiwan-headquartered Senhwa Biosciences Inc and the US National Institutes of Health (NIH) collaborated in analyzing the effectiveness of silmitasertib for the treatment of COVID-19 (https://www.biospectrumasia.com/news/34/15848/senhwa-biosciences-nih-to-co-develop-COVID-19-drug.html). The drug showed promise in controlling the proliferation of this RNA virus in human clinical tests. Silmitasertib was developed by Senhwa Biosciences to treat cancers, such as pediatric brain tumors, medulloblastoma, and bile duct cancer.

The second molecule tested was nintedanib, a tyrosine kinase inhibitor used to treat idiopathic pulmonary fibrosis or interstitial lung disease [40]. Very recently, the safety and efficacy of nintedanib ethanesulfonate have been analyzed in treating pulmonary fibrosis in patients with mild-to-extreme COVID-19. A placebo-controlled, a single-center, randomized study has been initiated and is currently in a phase 2 clinical trial (ClinicalTrials.gov identifier: NCT04338802).

In addition, the viral translation inhibitors ternatin and zotatifin, which is an FDA-approved drug for the treatment of multiple myeloma, have demonstrated the strongest binding affinity [41]. Plitidepsin is structurally similar to ternatin and is currently undergoing a clinical trial in COVID-19. The flavone luteolin, an antiinflammatory molecule, has broad antiviral properties [42, 43]. Previous studies have shown that luteolin inhibits SARS-CoV S protein and 3CL protease [44, 45]. Recently, both luteolin and quercetin have been identified through virtual screening and molecular docking as the best possible SARS-CoV-2 inhibitors [46, 47]. Furthermore, through SUMMIT, the world's most powerful supercomputer, high-throughput screening of small molecules interacting with the SARS-CoV-2 S protein or S protein–human ACE2 interface have recently been reported. Eriodictyol, a structural analog of luteolin, has been found to be a potential inhibitor of SARS-CoV-2 [48]. The last drug with considerable binding affinity toward N protein is fedratinib, an antiinflammatory JAK2 inhibitor. Wu et al. [49] have reported that fedratinib suppresses the expression of IL17, IL 22, and L23 in murine TH17 cells, and suggested that the drug may help mitigate the cytokine storm associated with SARS-CoV-2 infection. Stebbing et al. [50], through in silico artificial intelligence, have predicted significant beneficial effects of the antiinflammatory agents baricitinib, fedratinib, and ruxolitinib in the treatment of COVID-19.

The drug foretinib is an anticancer agent that inhibits vascular endothelial growth factor receptor (VEGFR) and hepatocyte growth factor receptor (HEGFR or MET) receptor. A recent study has reported that foretinib (DB12307) is a strong binder, on the basis of analysis through in silico virtual screening and molecular docking of 8548 ligands on the SARS-CoV-2 endoribonuclease NendoU (PDB ID: 6VWW) [51]. Our findings also have suggested that this drug binds the nucleocapsid protein with high binding affinity. Thus, foretinib may be repurposed as a broad-spectrum drug and tested against COVID-19 in the future.

Intriguingly, the docking of the N-NTD and C-CTD proteins revealed promising results for all five drugs tested. Notably, fedratinib and luteolin bind the ribonucleotide binding site and thus can inhibit RNA binding of the protein. Similarly, silmitasertib and nintedanib are positioned at the interface of two monomers and thus can impair the oligomerization of the protein. We recommend further experimental investigation of these compounds.

## Conclusion

The highly immunogenic and abundant nature of the N protein makes it a novel target to treat infection of the respiratory system by SARS-CoV2. We extended the investigation of drug efficacy, stimulated by the recent SARS-CoV-2-host interactome and identification of several anti-N drugs, by using computational analysis. In this study, binding modes were chosen, and the most common anti-N drugs were selected. The probable molecular underpinnings of their effectiveness against COVID-19 have been identified. The docking results indicated that 5 of 20 anti-N inhibitors bind with the energetic landscape of a protein–drug complex and have high thermodynamic scores. The identified drugs have been shown to bind the ribonucleotide binding pocket and protein interface of the N-NTD and C-CTD, thereby suggesting mechanisms of action. Thus, the identification of compounds that bind the N-NTD and C-CTD and interfere with NTD–RNA and NTD–NTD interactions may assist in the development of broad-spectrum antiviral therapeutics.

## Supplementary Information

Below is the link to the electronic supplementary material.

Supplementary file1 (DOCX 1371 kb)

## Data Availability

The data that support the findings of this study are available from the corresponding author upon reasonable request.
