# Supplementary material for: In Silico Identification of Potential Inhibitors of the SARS-CoV-2 Nucleocapsid Through Molecular Docking-Based Drug Repurposing
Source: Dr. Sulaiman Al Habib Med J. 2022 May 31;4(2):64–76. doi: 10.1007/s44229-022-00004-z (PMC9153216; doi:10.1007/s44229-022-00004-z)
Supplement: Supplementary file 1 — Supplementary file1 (DOCX 1371 kb) [file 44229_2022_4_MOESM1_ESM.docx]

***In silico identification of potential inhibitors for the Nucleocapsid of SARS-CoV-2 using molecular docking-based drug-repurposing***

Rukhsar Afreen^1#^, Saleem Iqbal^2#^, Ab Rauf Shah^3#^, Heena Afreen^4^, Lata Vodwal^5^, and Mohd. Shkir^6^*

*^1^Department of Zoology, Gargi College, University of Delhi, New Delhi-110049, India.*

*^2^Molecular Endocrinology and Nephrology, CHU Research Center and Laval University, Quebec City, Quebec, G1V 4G2, Canada.*

*^3^Department of Biochemistry, University of Nebraska-Lincoln, Nebraska 68503*

*^4^Department of Computer Science, Jamia Millia Islamia, New Delhi-110025, India.*

*^5^Department of Chemistry, Maitreyi College, Chanakyapuri, New Delhi.*

*^6^AFMOL, Department of Physics, College of Science, King Khalid University, Post Box- 9004, Abha-61413, Kingdom of Saudi Arabia.*

^#^ Authors having equal contribution

****Address for Correspondence***

**Corresponding Author**

**Mohd Shkir, Ph.D.**

**Associate Professor**

Email: [shkirphysics@gmail.com](mailto:shkirphysics@gmail.com)

Co-corresponding author

Email: [saleemiqbal550@gmail.com](mailto:saleemiqbal550@gmail.com)


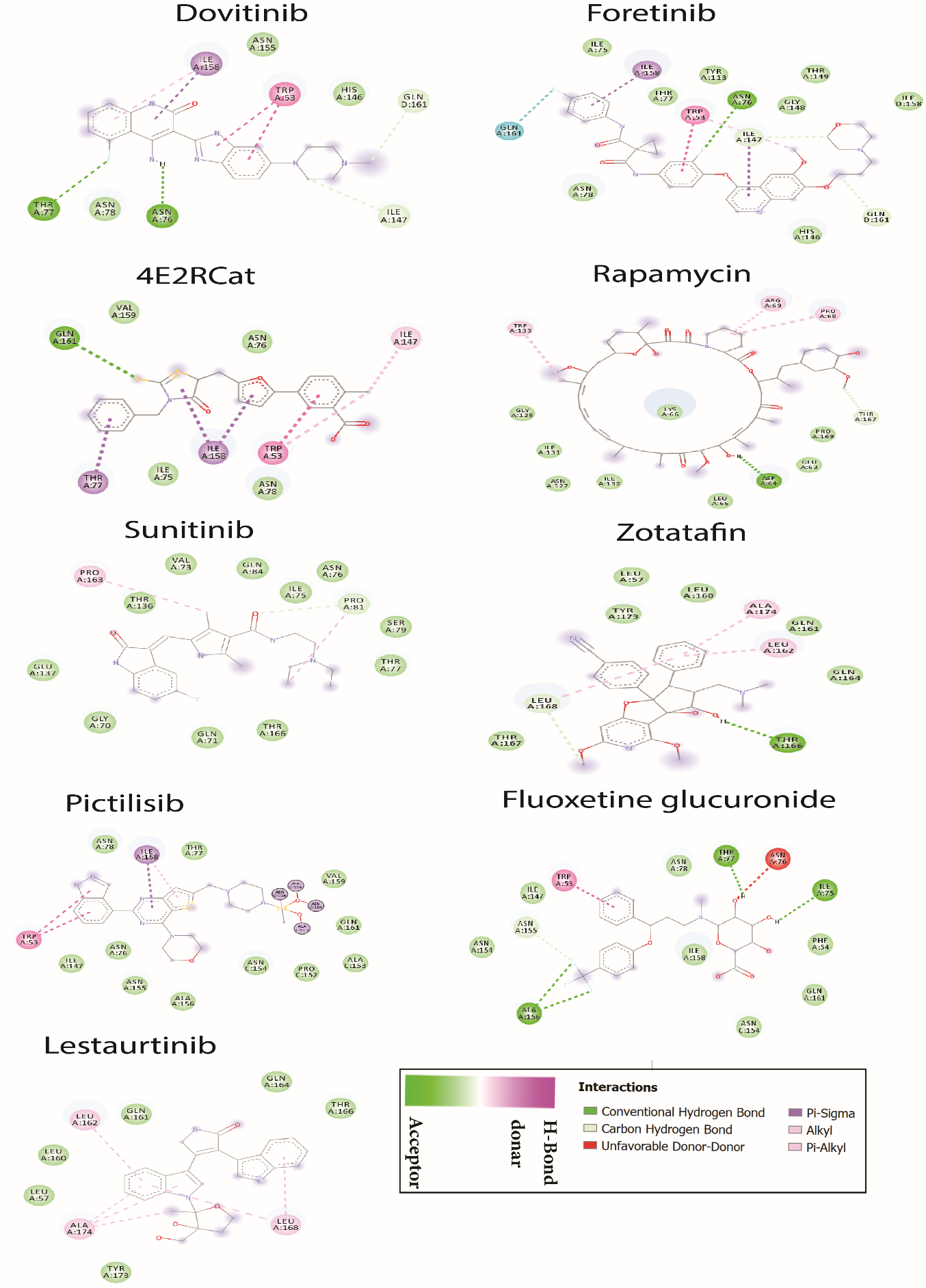


**Figure S1:** Molecular docking interactions and orientations of other anti-N drugs with SARS-CoV-2 N-NTD. The right panel shows schematic representation of interactions made by these drug molecules with N-NTD. The protein residues and interactions are colored accordingly and provided in figure.


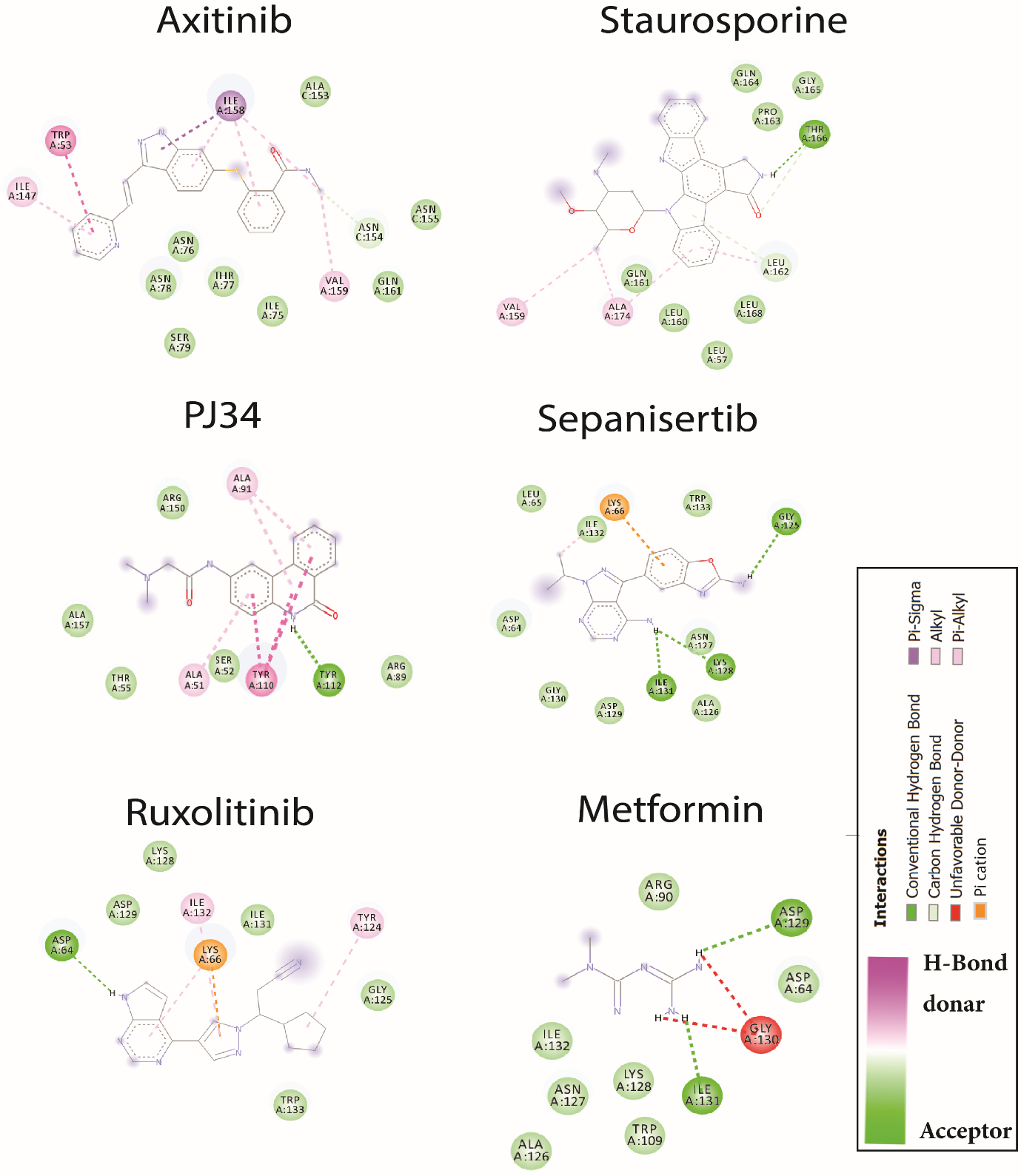


**Figure S2:** Molecular docking interactions and orientations of other anti-N drugs with SARS-CoV-2 N-NTD. The right panel shows schematic representation of interactions made by these drug molecules with N-NTD. The protein residues and interactions are colored accordingly and provided in figure.
